# Molecular Epidemiology of Novirhabdoviruses Emerging in Iranian Trout Farms

**DOI:** 10.3390/v13030448

**Published:** 2021-03-10

**Authors:** Sohrab Ahmadivand, Dušan Palić, Manfred Weidmann

**Affiliations:** 1 Department of Aquatic Animal Health, Faculty of Veterinary Medicine, University of Tehran, Tehran 1419963111, Iran; 2 Faculty of Veterinary Medicine, Ludwig-Maximilians University Munich, 80539 Munich, Germany; d.palic@lmu.de; 3 Institute of Microbiology and Virology, Medical School Brandenburg Theodor Fontane, 01968 Senftenberg, Germany

**Keywords:** VHS, IHN, glycoprotein (G) gene, haplotype analysis, phylogeny, trout

## Abstract

Novirhabdoviruses cause large epizootics and economic losses of farmed trout. In this study, we surveyed Viral hemorrhagic septicemia virus and Infectious hematopoietic and necrosis virus (VHSV and IHNV) through both monitoring and investigation of clinical outbreaks reported by farmers in the regions with major rainbow trout production in Iran from 2015 to 2019. RT-PCR assays of the kidney samples and cell culture (EPC/FHM cells) samples confirmed the presence of the viruses, with 9 VHSV and 4 IHNV isolates, in both endemic and new areas of Iran. Sequence analysis of the G gene revealed that VHSV isolates belonged to genogroup Ia, and IHNV isolates were clustered into genogroup E, both typical for isolates from European countries. A haplotype analysis based on non-homologous amino acids of the G gene supports the emergence of two lineages of IHNV from clade 1 (E-1), as well as VHSV clade 2 (Ia-2) of the European genogroups, confirming that VHSV and IHNV isolates in Iran, have originated from Europe possibly via imported eggs.

## 1. Introduction

Rhabdoviruses causing major epizootics in trout and salmon are OIE-listed Novirhabdoviruses Viral hemorrhagic septicemia virus, and Infectious hematopoietic and necrosis virus (VHSV and IHNV). Novirhabdoviruses carry an extra NV gene encoding a nonstructural protein of unknown function localized between the G and L genes [1,2] in the non-segmented negative ssRNA Rhabdovirus genome that generally encodes five structural proteins (N, P, M, G, and L) [3]. Five major genogroups of IHNV (E, J, L, M, and U), and four VHSV genogroups (I–IV) with several putative subgroups according to their geographic occurrence, have been described based on phylogenetic analysis of partial or complete G gene sequences [4,5,6]. The glycoprotein (G) is the protein responsible for attachment to the cell membrane receptors and induces neutralizing antibodies in infected fish. However, its high variability allows the viruses to evade host immune responses, persist in populations, and spread to new hosts and areas [4]. Genotyping of virus strains has confirmed the global spread of novirhabdoviruses due to aquaculture activities, specifically the eyed eggs trade [4,7,8].

Viral hemorrhagic septicemia virus (VHSV) infects more than 80 freshwater and marine species, while infectious hematopoietic necrosis (IHNV) has a limited number of known hosts and mainly affects younger fish [9,10]. Both VHS and IHN clinical disease outbreaks often occur at water temperatures below 15 °C (peak at 9–12 °C) and cause petechial hemorrhaging, dark skin, pale gills, exophthalmia, ascites, and/or erratic swimming with moderate to severe mortality (up to 100%) depending on the virus pathogenicity, host, and environmental conditions [9,10,11,12,13,14]. In general, IHN presents with fewer hemorrhagic symptoms than VHS [9,10].

Iran is one of the world-leading producers of freshwater rainbow trout (*Oncorhynchus mykiss*) with an annual production above 100 k tons and a respective annual demand for 300–400 million eyed eggs, of which 70% are imported from European countries [15]. Rainbow trout in Iran are reared in raceways, mainly in the Alborz and Zagros mountains, with cool summers and cold winters [15]. Viral diseases are the main problem of finfish aquaculture, with a high economic impact on the industry. However, commercial viral vaccines are not available in Iran [16,17]. Control and prevention of VHS and IHN in Iran thus depend upon surveillance schemes and biosecurity measures. VHSV and IHNV were first reported in Iran from farmed trout in late 2014, and sequence analyses indicated the European origin of the isolates [13,14]. While outbreaks of the viruses in Iranian trout farms are continuing, only a few studies investigated VHSV/IHNV and their genetic variants in two or three provinces before 2016 [18,19]. This study describes molecular characterization of VHSV and IHNV isolates obtained through monitoring activities and analyses of clinical outbreaks reported by farmers from 2015 to 2019.

## 2. Materials and Methods

### 2.1. Ethics Statement

All applicable guidelines for the care and use of animals by the University of Tehran Ethics Committee for Animal Experimentation in regards to diagnostic sampling were followed.

### 2.2. Fish Samples and Laboratory Examination

After the first report of VHSV and IHNV [13,14], the Iran National Veterinary Diagnostic Center (IVO) started a surveillance program in 2016 that covered aquaculture facilities throughout western, north-western, and central parts of Iran, based on the Office International des Epizooties (OIE) guidance [20,21], yielding isolates S.AV-IR-VHSV, S.AV-IR-VHSV1, S.AV-IR-VHSV2 in the first year. Additional isolates analyzed in this study were obtained from samples collected during clinical outbreaks reported 2015 to 2019 as suspected by farmers from Alborz, Mazandaran, Kurdistan, Kermanshah, Hamedan, Lorestan, Chaharmahal, and Bakhtiari provinces, where rainbow trout farming mostly takes place (Table 1). The surveyed fish farms were inland freshwater farms with average water temperature between 10 and 15 °C. In addition to the affected farm, three farms in each province were randomly selected, and 10 fish were sampled from each farm in all cases. After clinical examination, wet mounts of the gills and skin were prepared for parasitological examinations. Bacterial culture from the kidney tissue was performed on blood agar incubated at 24 °C for up to 72 h. Pools of kidney tissues from a maximum of five fish were used for virus isolation. Supernatants of positive cell cultures were tested by RT-PCR, and amplicons were sequenced in accordance with the standard OIE guidelines [20,21].

### 2.3. Virus Isolation

Kidney tissue samples were homogenized and inoculated in dilutions of 1:10 and 1:100 onto EPC and FHM cells in a 96-well plate, cultured in Minimal Essential Medium (MEM) supplemented with 10% FBS, l-glutamine, 100 IU penicillin G, and 100 μg/mL of streptomycin at 25 °C [22]. The inoculated cell cultures were incubated at 15 °C for two weeks and were examined daily for cytopathic effects (CPE). The supernatants of cultures developing positive CPE were sampled and processed for RT-PCR.

### 2.4. RT-PCR Assay

Kidney tissue samples (20 mg) were homogenized using a mortar and sterilized sand. The homogenate was diluted 1:10 in Hank’s balanced salts solution supplemented with 2% Fetal Bovine serum followed by centrifugation at 2500× *g* for 15 min at 4 °C. RNA was extracted directly from the homogenates or 150 mL virus supernatant using the RiboEx SL Total RNA extraction and Exgene^TM^ Viral DNA/RNA kits (respectively), following the manufacturer’s instructions (GeneAll, Seoul, Korea). The cDNA synthesis was carried out with 5 μL of the extracted RNA using the HyperScript^TM^ First strand Synthesis Kit (GeneAll, Korea) according to the manufacturer’s instructions in a total volume of 25 µL. The samples were screened for VHSV and IHNV using VHSV primers VHS-F (5′GCATGCACAGTGACATTCTG, and VHS-R (5′GAGCATTCCACTGTCATAGAC), and OIE recommended IHNV primers IHN-F (5′-AGAGATCCCTACACCAGAGAC-3′) and IHN-R (5′-GGTGGTGTTGTTTCCGTGCAA-3′) yielding 513 bp and 693 bp Glycoprotein (G) gene amplification products, respectively. The PCR assay was performed in a final volume of 50 μL containing 25 μL Taq DNA Polymerase Mix Red (GeneAll, Korea), 4 μL of cDNA, 1.5 μL of each primer pair (10 pmol), and 18 μL nuclease-free water. PBS was used as a negative control instead of the virus genome in PCR assay. The PCR was performed using the following temperature profile: denaturation at 10 min 95 °C followed by 35 cycles of 94 °C for 30 s, 52 °C (IHNV) or 58 °C (VHSV) for 35 s and 72 °C for 45 s, and a final extension at 72 °C for 10 min. Samples were additionally screened for IPNV using PCR assays by previously published primers with an amplicon of 405 bp region of the VP2 gene, according to Ahmadivand et al. [23]. The amplification products were resolved with electrophoresis using a 1.5% agarose gel and visualized under UV light. Furthermore, PCR products were purified using Expin^TM^ PCR SV kit (GeneAll, Korea), and then both cDNA strands were sequenced using a commercial sequencing provider (Bioneer Co., Daejeon, Korea).

### 2.5. Sequence Analysis 

Nucleotide sequences were analyzed using Geneious Prime (www.geneious.com (accessed on 6 March 2021)) and the BioEdit software [24], and the National Centre for Biotechnology Information (NCBI) BLAST tool. The neighbor-joining method and the HKY model of nucleotide substitution using 1000 bootstrap replicates were performed for phylogenetic tree construction [25]. SPLITS TREE 5.0 [26], was used for Neighbor network and Parsimony network analysis of the IHNV (303 bp Mid G gene) and VHSV (693 bp) G proteins after deleting homologous amino acid columns from a ClustalW alignment in MEGA6 [27], resulting in a 29- and a 35-character set for the glycoprotein amino acid sequence of VHSV and IHNV, respectively.

## 3. Results

### 3.1. Outbreaks Description

From May 2015 to November 2019, the causative agents of 13 outbreaks of infectious disease in rainbow trout farms were diagnosed as caused by VHSV and IHNV. Altogether 9 VHSV and 4 IHNV isolates were obtained (Table 1). Cumulative mortalities of outbreaks at 10–15 °C were estimated at 20–70% based on information obtained from farmers during farm visits. Mortality records were not available. Trout fry (<10 g) were primarily affected by IHNV, while VHSV outbreaks occurred in larger fish (10–400 g) (Table 1). The clinical signs and gross pathology of infected fish were similar to those previously described for VHSV [9] and IHNV [10]. The fish samples showed no significant parasite loads, and pathogenic bacteria were not isolated.

### 3.2. Viral Isolation and RT-PCR Assay

**Figure 1:**
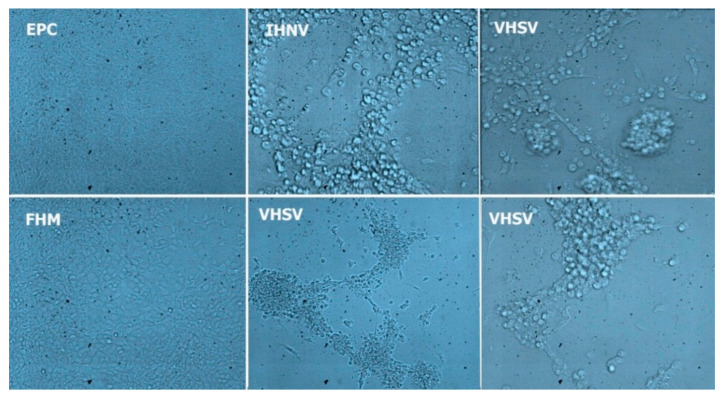
Cytopathic effect (CPE) caused by Viral hemorrhagic septicemia virus and Infectious hematopoietic and necrosis virus (VHSV and IHNV) in EPC and FHM cell lines at different days post-inoculations (dpi). The upper row shows control uninfected EPC cells (40×), IHNV, and VHSV infected EPC cells showing CPE at 5 dpi (100×). The bottom row shows FHM control cells (40×) and infected FHM cells with VHSV at 5 dpi (40× and 100×, respectively).

**Figure 2:**
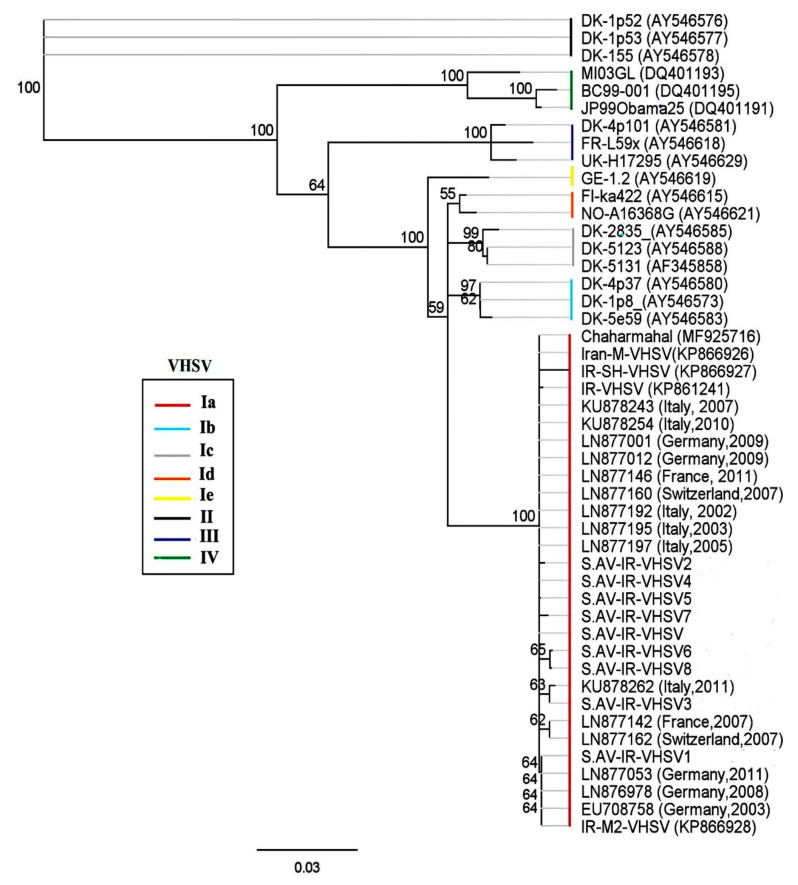
Phylogenetic analysis of Iranian isolate of VHSV based on the partial nucleotide sequences of G gene (513 bp). The phylogenetic trees were constructed using the Geneious Prime (Neighbor-joining with the Hasegawa-Kishino-Yano (HKY) model and 1000 bootstrap replicates). The Iranian isolates of VHSV (S.AV-IR-VHSV to S.AV-IR-VHSV8) were classified as an Ia genogroup (European origin). GenBank accession numbers are listed in Table 1.

Four kidney tissue filtrates generated from pooled samples collected from farms during the 2016 surveillance induced CPE in EPC and FHM cell lines (VHSV: S.AV-IR-VHSV, S.AV-IR-VHSV1, S.AV-IR-VHSV2, and IHNV: S.AV-IR-IHNV1). The EPC was characterized by rounded and granular cell grape-like clusters (Table 1 and Figure 1). The VHSV and IHNV isolates induced the typical novirhabdovirus CPE in EPC and FHM cell lines in the first passage 2–5 days post-inoculation (Figure 2). RT-PCR screening of CPE positive cell culture supernatants produced specific 513 bp and 693 bp fragments for VHSV and IHNV G gene, respectively. In addition, RT-PCR screening of pooled kidney samples from nine farms revealed VHSV (in six farms) and IHNV (in three farms) specific amplicons (data not shown). All VHSV or IHNV positive samples were IPNV-VP2-PCR negative.

### 3.3. Sequence Analysis

#### 3.3.1. VHS

The amplicons were verified by sequencing, and the determined sequences were deposited in the NCBI GenBank (Table 1). In alignments, the partial G sequences of the VHSV isolates showed high nucleotide and amino acid identity (>99%) among themselves and with previously reported Iranian isolates (KP866928, KP866927, KP866926, MF925716). Moreover, the sequences revealed the closest identity (up to 100%) to sequences of Italian (KU878254, LN877197, KU878243, KU878262, LN877192, LN877195) and German isolates (EU708758, LN877001, LN877053, LN876978, LN877012), as well as sequences of isolates from France (LN877146, LN877142), and Switzerland (LN877160, LN877162), originating from farmed freshwater trout.

**Figure 3:**
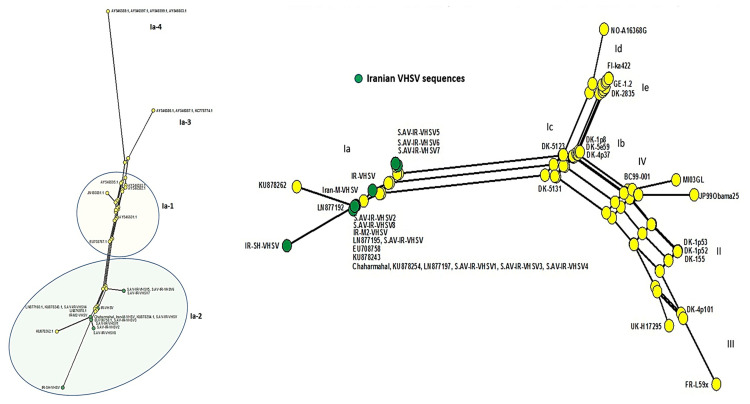
Neighbor network of non-homologous amino acids of VHSV glycoprotein fragment (513 bp). Left panel genotype Ia sequences only. Right panel all genotype sequences as indicated in Figure 2. Green circles indicate Iranian sequences.

Phylogenetic analysis using VHSV-G sequences derived from outbreaks in Iranian trout farms, as well as representative sequences from all four genogroups, revealed that the detected isolates belong to the genogroup Ia (European origin) (Figure 2). Moreover, subclades within the genogroup were formed by Iranian isolates S.AV-IR-VHSV6 (MT431659), S.AV-IR-VHSV8 (MT431657), and S.AV-IR-VHSV1 (KX609424) with the German isolate (LN877053), and by S.AV-IR-VHSV3 (MK279321) with the Italian isolate (KU878262). The phylogenetic relationship between the Ia clades revealed that all the Iranian VHSV sequences belonged to clade Ia-2 (Supplementary Figure S1). To analyze the relationship of the subclades in more detail, a haplotype analysis based on non-homologous amino acids of G was performed. The resulting network supported the emergence of subclade 2 from clade Ia (Figure 3).

#### 3.3.2. IHNV

Sequence analysis of the partial G gene sequences (693 bp) of Iranian IHNV isolates from trout hatcheries (Table 1) showed high identity (>99%) among themselves and with those previously reported in Iran (KX756440, KR814574, MG753776, and MG753777). The isolates also displayed the highest sequence identities (>99%) with Italian isolates (KU878324, KU878336, KU878353, KU878357, KU878359, and FJ711511), as well as sequences of isolates from Switzerland (LN897491) and Germany (LN897533, LN897542, and LN897560).

**Figure 4:**
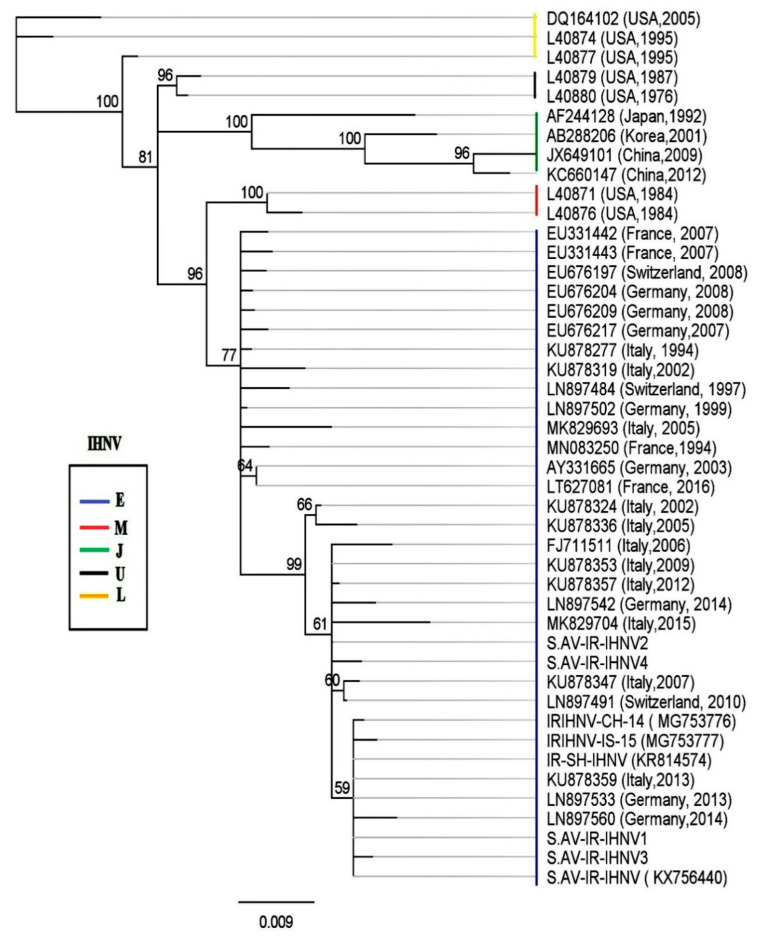
Phylogenetic analysis of Iranian isolate of IHNV based on the partial nucleotide sequences of G gene (693 bp). The phylogenetic trees were constructed using the Geneious Prime (Neighbor-joining with the HKY model and bootstrap replicates of 1000). The Iranian isolate of IHNV (S.AV-IR-IHNV1- S.AV-IR-IHNV4) was classified as a European (E) genogroup (GenBank accession numbers are listed in Table 1).

**Figure 5:**
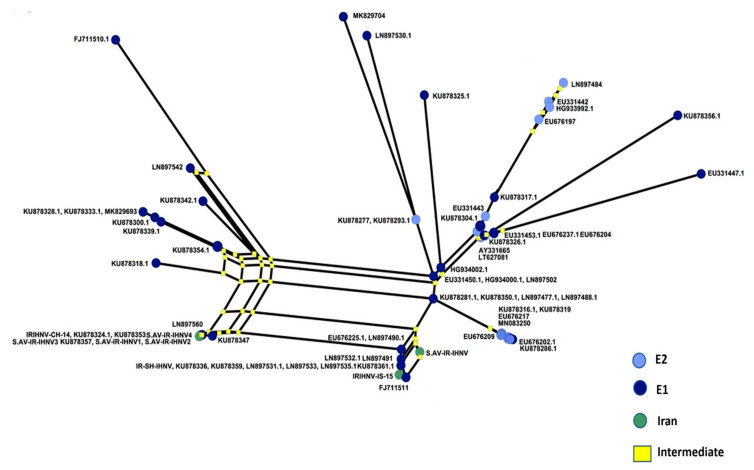
Neighbor network of non-homologous amino acids of IHNV glycoprotein fragment (303 bp mid G gene). IHNV sequences as indicated in Figure 4. Green circles indicate Iranian sequences.

Phylogenetic analysis revealed that all the Iranian IHNV isolates belong to the European genogroup (E), with a bootstrap value of 96 (Figure 4). Moreover, S.AV-IR-IHNV1 (MK279324) and S.AV-IR-IHNV3 (MT431656) clustered together with previously described Iranian isolates, as well as published sequences from Germany (LN897533 and LN897560), and Italy (KU878359), supported by an inner clade bootstrap value of 59. Again, a haplotype analysis based on non-homologous amino acids of G was performed, and the resulting network indicated two lineages of IHNV originating from clade 1 (E-1) of the European genogroup (Figure 5).

## 4. Discussion

Novirhabdoviruses (IHNV and VHSV) can cause epizootics and substantial economic losses for the trout industry [4]. It is, therefore, of major importance to implement novirhabdovirus surveillance programs and epidemiological studies in the farmed trout populations to facilitate early detection, effective health management, and eradication [6]. Since the first report for these viruses from Iran, a few publications have described their detection in two or three provinces before 2016 [13,14,18,19]. 

To better understand virus origins and spreading patterns, both IHNV and VHSV in Iranian trout farms were surveyed in 2016, and samples were collected from farms reporting suspected outbreaks from May 2015 to November 2019. Our study revealed that VHSV/IHNV outbreaks are continuing in some Iranian provinces. The first outbreak of IHNV was detected in Lorestan, and the first outbreaks of VHSV were detected in Kurdistan, Kermanshah, Lorestan, Alborz, and Tehran provinces. Virus isolation was attempted and confirmed by RT-PCR and sequencing of the amplicons. Clinical signs reported in previous studies were again observed in infected fish of this study, whereas estimated mortality rates were inconsistent at 20–70% at different farms. Young trout showed higher mortalities than older fish, indicating the essential role of age in the susceptibility to VHSV and IHNV infections [9,10,11,12,13,14].

Novirhabdoviruses are easily grown on cell monolayers with typical lesions, and molecular-based or antibody-based identification is recommended diagnostic method for these viruses [20,21]. EPC cells are the most susceptible to IHNV [22], and VHSV produces high titers in this cell line [28]. In terms of permissiveness to VHSV, fish cell lines can be ranked as follows: BF-2, FHM, RTG-2, and EPC [29]. EPC cell lines have been shown to be more susceptible to VHSV genotype IV isolates than to type I to III isolates [30]. Earlier it had been shown that FHM cells show the greatest variability in terms of sensitivity to novirhabdovirus isolation across laboratories [22]. Nonetheless, in our study, EPC and FHM cell lines showed no differences in terms of VHSV culture, which could be due to the particular FHM cell line used in our laboratory. In addition, in recent years, FHM cells have increasingly been used as a model cell line for VHSV pathogenesis research [31,32]. 

Previous surveys of Iranian trout farms reported only a few detections of the viruses when pooled organ samples were tested in cell culture isolation using the CHSE-214 cell line [18,19]. Compared to our results, it appears that using less susceptible CHSE-214 cells for diagnostic isolation of VHSV and IHNV may have contributed to underreporting. Additionally, it is well demonstrated that RT-PCR is more sensitive than cell culture in detecting the presence of viral genetic material [33,34]. An RT-PCR assay of pooled samples was, therefore, the main choice of method used in this study to detect low viral titers in fish.

Sequencing based on the G gene revealed that the nine VHSV sequences belonged to genotype Ia-2, and four IHNV sequences clustered with genotype E-1, which were characterized by isolates from trout farms from European countries. Previous investigations of IHNV or VHSV from Iran have also detected these genotypes with close identity [14,18,19], suggesting the European origin of the viruses and their possible introduction to Iran through imported eyed eggs [7,8,14]. True vertical transmission of VHSV and IHNV is unlikely to occur [8,16]. However, inadequate external disinfection of eggs from infected broodstock can contribute to the spread of IHNV [16]. The annual demand of 300–400 million eyed eggs for the Iranian aquaculture industry is met by more than 70% via imports from European countries [15]. However, due to the economic and political situation, some of this import is presumably not under proper veterinary control. Moreover, Iranian fish farms are tightly interconnected and exchange eggs, small fry, and fish each year providing ample opportunity for the spread of the viruses across the sector. Indeed, frequent exchange of the viruses between farms might be the main reason for the close identity of the Iranian isolates with each other.

A recent study from Finland has shown that voluntary submission of suspected diseased fish by farmers is a more efficient approach to surveillance compared to government-based surveillance programs [35]. Therefore, the aquaculture industry in Iran should find ways of sponsoring this type of proactive voluntary and industry-driven surveillance program, which yielded the majority of isolates described here, and it needs to review and standardize biosecurity measures in the production of farmed trout to better control the continued spread of VHSV and IHNV across the sector.

In conclusion, our study provides evidence for outbreaks of the European genotypes of IHNV and VHSV possibly introduced via imported eggs, associated with 13 mortality outbreaks in more regions of Iran than previously known from 2015 to 2019. Considering the importance of the trout industry in Iran, improvement in biosecurity measures and the development of effective vaccines are also highly recommended.

## Figures and Tables

**Table 1:** Details of VHSV and IHNV outbreaks in farmed rainbow trout (*O. mykiss*) included in this study.

Isolates	Province/City	Month/Year	Virus	Fish Size (g)	Mortality ^1^	Source ^2^	Acc. No
S.AV-IR-VHSV	Mazandaran/Amol	June 2016	VHSV	120–150	20–30%	EPC-cell culture	KX609423
S.AV-IR-VHSV1	Mazandaran/Amol	February 2016	VHSV	20–30	50–70%	FHM-cell culture	KX609424
S.AV-IR-VHSV2	Hamedan/ Nahavand	May 2016	VHSV	80–100	40–50%	EPC-cell culture	MK279320
S.AV-IR-VHSV3	Kurdistan/ Kamyaran	April 2017	VHSV	50–60	30–40%	Kidney tissue	MK279321
S.AV-IR-VHSV4	Mazandaran/Amol	May 2015	VHSV	10–30	60–80%	Kidney tissue	MK279322
S.AV-IR-VHSV5	Chaharmahal and Bakhtiari/ShahreKord	December 2015	VHSV	100–140	<30%	Kidney tissue	MK279323
S.AV-IR-VHSV6	Kermanshah/Paveh	January 2018	VHSV	200–250	20–30%	Kidney tissue	MT431659
S.AV-IR-VHSV7	Alborz/Karaj	August 2019	VHSV	40–60	30–40%	Kidney tissue	MT431658
S.AV-IR-VHSV8	Lorestan/Aleshtar	September 2019	VHSV	300–400	<20%	Kidney tissue	MT431657
S.AV-IR-IHNV1	Tehran/Firuzkuh	March 2016	IHNV	1–2	60–70%	EPC-cell culture	MK279324
S.AV-IR-IHNV2	Chaharmahal and Bakhtiari/Boroujen	February 2017	IHNV	5–10	20–30%	Kidney tissue	MK279325
S.AV-IR-IHNV3	Hamedan/ Nahavand	October 2019	IHNV	1–2	40–60%	Kidney tissue	MT431656
S.AV-IR-IHNV4	Lorestan/Aleshtar	November 2019	IHNV	1–5	<30%	Kidney tissue	MT431655

^1^ Crude mortality (%) estimates as reported by farmers at water temperature of 10 °C to 15 °C. ^2^ RNA source for RT-PCR diagnostic (cell culture supernatant or homogenized kidney tissue).

## Data Availability

Not applicable.

## Supplementary Materials

The following are available online at https://www.mdpi.com/1999-4915/13/3/448/s1, Figure S1: The phylogenetic analysis of the Ia clades of VHSV isolates based on the partial nucleotide sequences of G gene (513 bp).
