# Supplementary material for: Molecular Epidemiology of Novirhabdoviruses Emerging in Iranian Trout Farms
Source: Viruses. 2021 Mar 10;13(3):448. doi: 10.3390/v13030448 (PMC7999222; doi:10.3390/v13030448)
Supplement: Supplementary file 1 [file viruses-13-00448-s001.pdf]

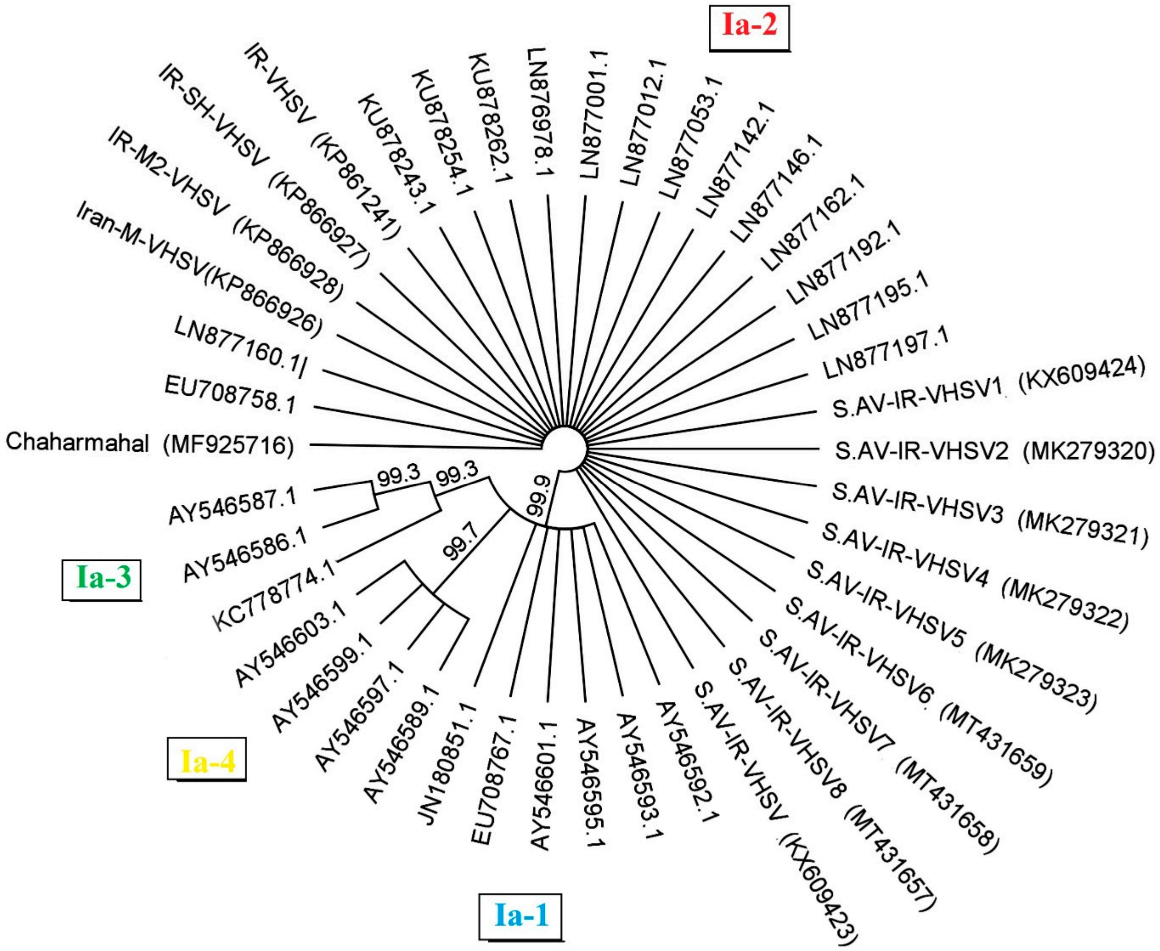

Supplementary Figure S1. The phylogenetic analysis of the Ia clades of VHSV isolates based on the partial nucleotide sequences of G gene (513 bp). The phylogenetic trees were constructed using the Geneious Prime (Neighbor-joining with the HKY model, and 1000 bootstrap replicates). The Iranian isolate of VHSV (S.AV-IR-VHSV to S.AV-IR-VHSV8) were classified as subclade 2 from clade Ia (Ia-2; European origin).
